# Automated histological classification of whole slide images of colorectal biopsy specimens

**DOI:** 10.18632/oncotarget.21819

**Published:** 2017-10-12

**Authors:** Hiroshi Yoshida, Yoshiko Yamashita, Taichi Shimazu, Eric Cosatto, Tomoharu Kiyuna, Hirokazu Taniguchi, Shigeki Sekine, Atsushi Ochiai

**Affiliations:** ^1^ Division of Pathology and Clinical Laboratories, National Cancer Center Hospital, Chuo-ku, Tokyo, Japan; ^2^ Medical Solutions Division, NEC Corporation, Minato-ku, Tokyo, Japan; ^3^ Epidemiology and Prevention Group, Research Center for Cancer Prevention and Screening, National Cancer Center, Chuo-ku, Tokyo, Japan; ^4^ Department of Machine Learning, NEC Laboratories America, Princeton, NJ, USA; ^5^ Division of Molecular Pathology, National Cancer Center Research Institute, Chuo-ku, Tokyo, Japan; ^6^ Division of Pathology, Research Center for Innovative Oncology, National Cancer Center, Kashiwa, Chiba, Japan

**Keywords:** colorectal cancer, artificial intelligence, automated image analysis, whole slide imaging, histological classification, Pathology Section

## Abstract

**Background:**

An automated image analysis system, e-Pathologist, was developed to improve the quality of colorectal biopsy diagnostics in routine pathology practice.

**Objective:**

The aim of the study was to evaluate the classification accuracy of the e-Pathologist image analysis software in the setting of routine pathology practice in two institutions.

**Materials and methods:**

In total, 1328 colorectal tissue specimens were consecutively obtained from two hospitals (1077 tissues from Tokyo hospital, and 251 tissues from East hospital) and the stained specimen slides were anonymized and digitized. At least two experienced gastrointestinal pathologists evaluated each slide for pathological diagnosis. We compared the 3-tier classification results (carcinoma or suspicion of carcinoma, adenoma, and lastly negative for a neoplastic lesion) between the human pathologists and that of e-Pathologist.

**Results:**

For the Tokyo hospital specimens, all carcinoma tissues were correctly classified (n=112), and 9.9% (80/810) of the adenoma tissues were incorrectly classified as negative. For the East hospital specimens, 0 out of the 51 adenoma tissues were incorrectly classified as negative while 9.3% (11/118) of the carcinoma tissues were incorrectly classified as either adenoma, or negative. For the Tokyo and East hospital datasets, the undetected rate of carcinoma, undetected rate of adenoma, and over-detected proportion were 0% and 9.3%, 9.9% and 0%, and 36.1% and 27.1%, respectively.

**Conclusions:**

This image analysis system requires some improvements; however, it has the potential to assist pathologists in quality improvement of routine pathological practice in the not too distant future.

## INTRODUCTION

Colorectal cancer is a major cause of morbidity and mortality in Japan, where it is the leading cause of death among women, and the third leading cause of death among men (from malignant neoplasms) [1]. A large number of specimens is therefore obtained with endoscopes, in order for pathologists to make their respective diagnoses. Moreover, in recent years, double-checking of slides by pathologists has been widely recommended to ensure correct diagnosis. In contrast, while the number of pathologists has remained essentially constant, the number of cases has increased, and therefore the diagnostic burden on pathologists has grown.

Under these circumstances mentioned above, histological image analysis methods have been proposed for colorectal cancer detection [2-6]. Esgiar et al. [2] performed classification of normal and cancerous classes based on texture analysis techniques, and reported the classification result on a dataset that consists of 44 normal and 58 cancer images captured using a microscope with a 40x objective lens, and 512x512 image resolution. Altunbay et al. [3] provided a method based on graph algorithm, and reported the classification result on a test image set consisting of 34 normal specimens, 35 low-grade cancerous specimens, and 29 high-grade cancerous specimens, captured by a microscope at 20x magnification, with a resolution of 480x640 pixels. Jiao et al. [4] performed class discrimination based on statistical texture analysis using the Gray-Level Co-occurrence Matrix method [7], and reported the discrimination result on a dataset consisting of 30 normal core images, and 30 abnormal core images cropped from a whole slide image (WSI), which is a Tissue Microarray slide consisting of 60 core arrays, scanned by a digital slide scanner. Kalkan et al. [5] reported the results of categorization of small patch images at 1024x1024 dimensions into four categories: normal, cancer, adenomatous, and inflamed classes on a dataset consisting of 2000 image patches per category, cropped from 55 WSIs of 36 patients. Ozdemir et al. [6] proposed a resampling-based Markovian model, and showed experiments of classifying a test set of 258 patients consisting of 491 normal, 844 low-grade cancerous, and 257 high-grade cancerous images taken with a microscope using a 20x microscope objective lens with 480x640 image resolution.

In reviewing previous studies, emphasis has been placed on the methods of image analysis, rather than a practical computer-assisted diagnosis system for clinical use. Furthermore, the efficacy of these methods was only evaluated on a small amount of data, and many previous studies also only focused on small images photographed by a microscope. Although there are a few studies that addressed WSIs, these studies focused on only limited, manually selected regions of tissues slides rather than the entire WSI of each slide. The region selection processes involving manual operation have ensured higher quality of input images, but also decreased computational complexity. In practice, it is desirable that a computer-assisted diagnosis system be fully automated, and therefore free from operators. Unfortunately, however, literature on the topic is relatively sparse.

The co-authors at the NEC Corporation have developed an automated pathological image analysis system for colorectal biopsy specimens, named “e-Pathologist*”*. This system detects abnormal tissue suggestive of malignancy on hematoxylin and eosin (H&E)-stained sections and gives alerts to the user (i.e. pathologists) to reduce the risk of incorrect diagnoses. This system automatically detects and analyses WSI files generated by a digital slide scanner. The general flow of this system is as follows: This system distinguishes between adjacent tissues in the whole image, and recognizes each tissue as an individual entity. Each tissue region is then cropped from the WSI at 20x magnification (average image size is 10,000x10,000 pixels). Each tissue image is further analyzed and classified as four categories: suspicious for carcinoma, suspicious for adenoma, no neoplasia, and unclassifiable.

The aim of this study was to evaluate the classification performance of the proposed system using samples collected for routine diagnostics, from two institutions. In addition, we assessed its effectiveness and explored the feasibility of using the system routinely as part of modern clinical practice.

## RESULTS

Our experiment was performed with datasets from two institutions under routine conditions. Test tissues were composed of 1077 tissues from Tokyo hospital, and 251 tissues from East hospital. Table 2 shows the classification result table, confusion matrix, in the Tokyo hospital, and East hospital datasets. Here, the classifier had been trained using training set, and the parameters tuned in advance using the validation set, so that an undetected rate carcinoma would be less than 1%, and an undetected rate of adenoma would be less than 5%. Note that the facilities providing both the training set and the validation set were different from the facilities providing the two test sets, which are target data in this study.

**Table 1:** Table defining the similarities between the revised Vienna classification, Japanese group classification, and classification of our system

The revised Vienna classification	Description	Japanese group classification	classification by our system
Category 1	Negative for neoplasia/dysplasia	Group 1	Negative
Category 2	Indefinite for neoplasia/dysplasia	Group 2	Negative
Category 3	Low grade adenoma/dysplasia	Group 3	Adenoma
Category 4.1	High grade adenoma/dysplasia	Group 4	Positive
Category 4.2	Non-invasive carcinoma (Ca in situ)	Group 5	Positive
Category 4.3	Suspicion for invasive carcinoma	Group 5	Positive
Category 5.1	Intramucosal carcinoma	Group 5	Positive
Category 5.2	Submucosal carcinoma or beyond	Group 5	Positive
Category X	Inadequate specimen for diagnosis	Group X	unclassifiable

**Table 2:** Confusion matrix in the Tokyo hospital, and East hospital datasets

			system											
			Tokyo hospital						East hospital					
			P	A	N	X	total		P	A	N	X	total	
**Human pathologist**	**P**	**Group 5**	112	0	0	0	112	10.4%	107	8	3	0	118	47.0%
		**lymphoma**	8	3	6	0	17	1.6%	5	0	0	0	5	2.0%
		**carcinoid**	0	0	0	0	0	0.0%	1	0	1	0	2	0.8%
		**Group 4**	0	0	0	0	0	0.0%	0	0	0	0	0	0.0%
	**A**	**Group 3**	338	385	80	7	810	75.2%	25	26	0	0	51	20.3%
	**N**	**Group 2**	0	0	0	0	0	0.0%	0	0	1	0	1	0.4%
		**Group 1**	16	28	94	0	138	12.8%	12	31	31	0	74	29.5%
	**X**	**Group X**	0	0	0	0	0	0.0%	0	0	0	0	0	0.0%
	**total**		474	416	180	7	1077	100.0%	150	65	36	0	251	100.0%

P = Positive, A = Adenoma, N = Negative, X = Unclassifiable

As shown in Table 2, for the Tokyo hospital dataset, the 112 Group 5 tissues were fully correctly classified while the 80 out of the 810 Group 3 tissues were under-classified as Negative(N). In contrast, for the East hospital dataset, none of the 51 Group 3 tissues were under-classified as Negative(N) while the 11 out of the 118 Group 5 tissues were under-classified as Adenoma(A), or Negative(N). For the Tokyo hospital dataset, the undetected rate of carcinoma, undetected rate of adenoma, and over-detected proportion are 0.0%, 9.9%, and 36.1%, respectively. For the East hospital dataset, the undetected rate of carcinoma, undetected rate of adenoma, and over-detected proportion are 9.3%, 0.0%, and 27.1%, respectively. The summary of classification performances is shown in Table 3 together with the system performance, which tuned parameters by using the validation data as the reference value for comparison.

**Table 3:** Classification performances

	undetected rate of carcinoma	undetected rate of adenoma	over-detected proportion
**Tokyo hospital**	0.0%	9.9%	36.1%
**East hospital**	9.3%	0.0%	27.1%
**performance tuned by using validation data**	≈1%	≈5%	≈30%

## DISCUSSION

The present study evaluated the automated pathological image analysis system (e-Pathologist) for colorectal specimens with the goals of clarifying scope and requirements for putting the system into practical use. Amid the changing environment surrounding pathology division, utilizing such a system is one possible solution to be able to efficiently make a diagnosis. However, no image analysis system has been tested with a large dataset of patients, in routine conditions, and multi institutions. This is the first study to evaluate the classification performance of the proposed system using the datasets collected from two institutions in routine diagnosis condition and explore the practical use of this system as considering the advantages along with the disadvantages.

**Figure 1:**
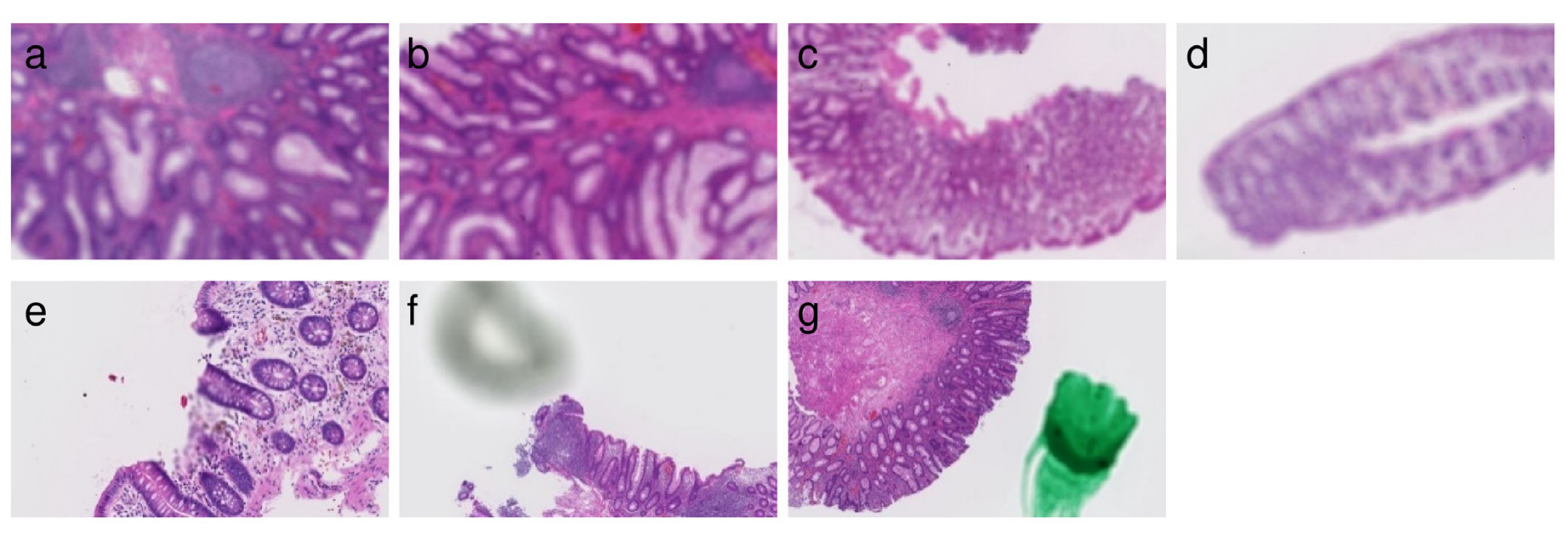
Seven tissues classified as Unclassifiable(X) The following images are excerpts from the tissue images. (a)-(d) Overall blurred images. (e) Partial blurred image. (f) Image with shadow close by a tissue. (g) Image with a pen mark.

Evaluation results are summarized in the confusion matrices (Table 2). In Tokyo hospital, the seven tissues shown in Figure 1 were classified as Unclassifiable(X). The cause of being Unclassifiable(X) is found to be blurred regions, shadows, and pen marks. Therefore, it has been confirmed that this system provided the functions to properly exclude such artifacts.

**Figure 2:**
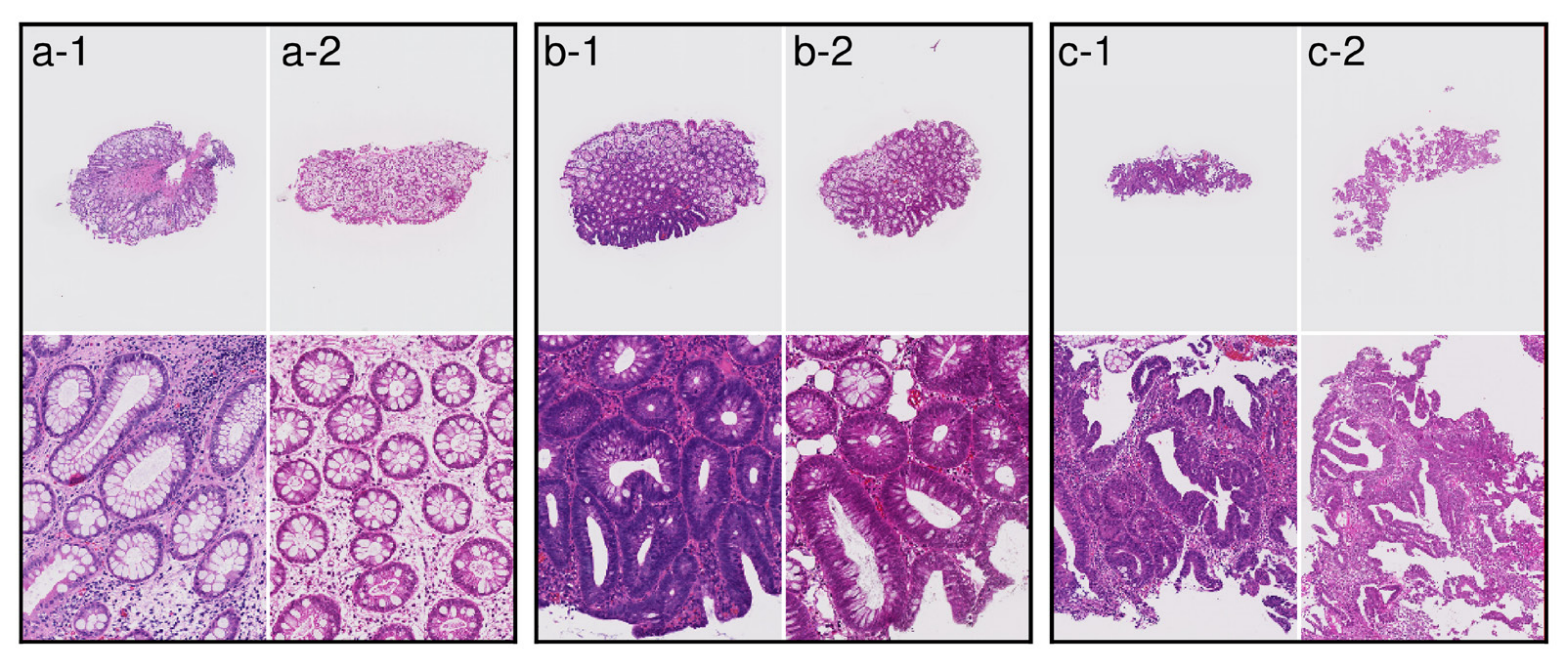
Examples of tissue images from Tokyo hospital and East hospital (a-1), (b-1), and (c-1) Examples of tissue images at Tokyo hospital. (a-2), (b-2), and (c-2) Examples of tissue images at East hospital. Differences in slide preparation between two batches give rise to differences in image colors.

**Figure 3:**
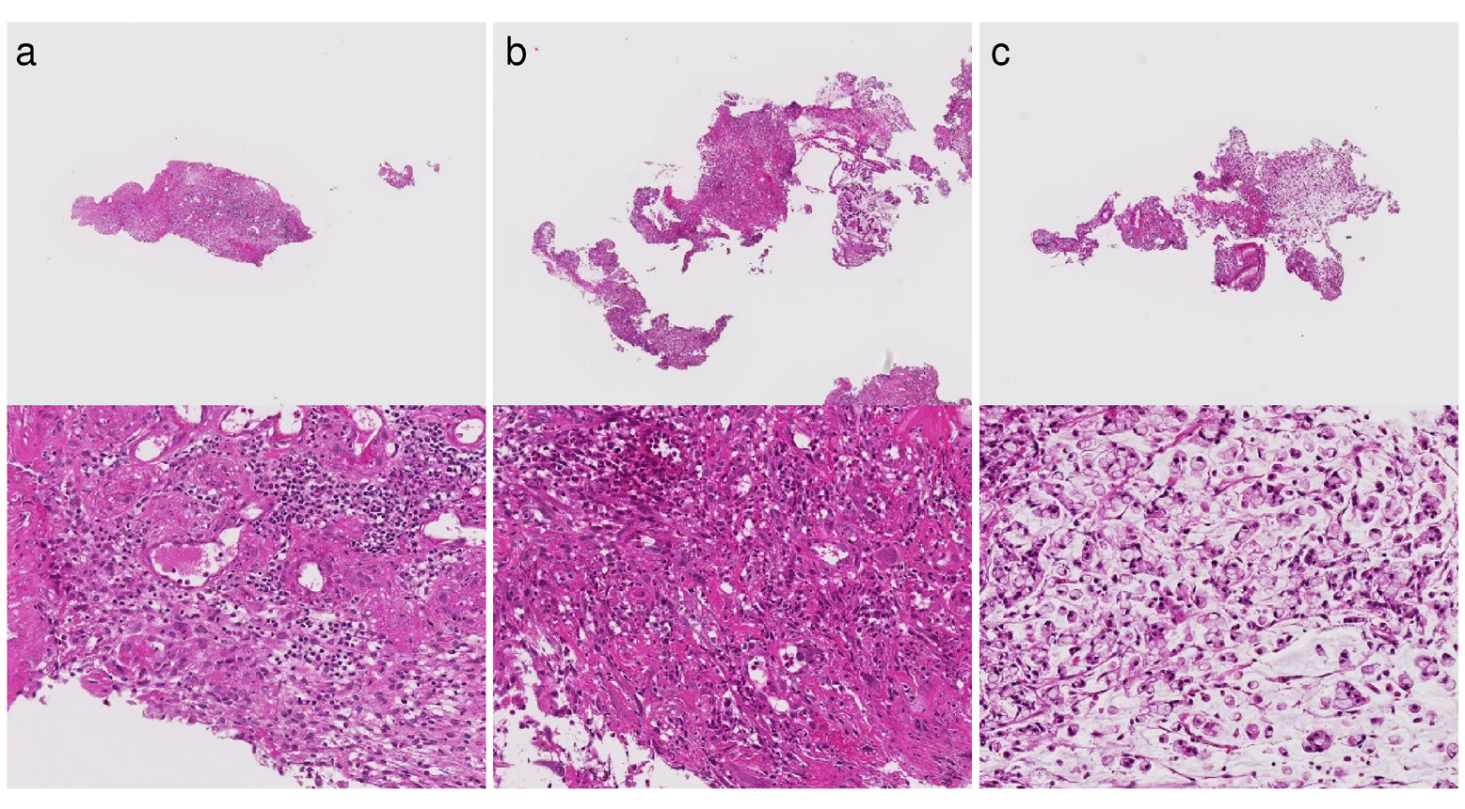
Three tissues classified as Negative(N) in case a diagnosis by human pathologists is Group 5 on East hospital dataset (a) Poorly differentiated adenocarcinoma. (b) Poorly differentiated adenocarcinoma. (c) Signet-ring cell carcinoma.

The representative tissue images from two hospitals are shown in Figure 2. We speculate that these color differences were largely caused by H&E staining conditions rather than by the individual differences among the scanners at each institution. Differences in slide preparation between two batches give rise to differences in image properties. Histopathological image analysis often suffers from batch effects of color tone and color contrast. All of the Group 5 tissues were fully correctly classified on the Tokyo hospital dataset, while the 3 out of 118 Group 5 tissues were under-classified as Negative(N) on the East hospital dataset. The three tissue images are shown in Figure 3. The two out of three tissues were poorly differentiated adenocarcinoma, and the other tissue was signet-ring cell carcinoma. The poorly differentiated adenocarcinoma was classified based on cytological atypia analysis, where nuclei were extracted. In the tissue images with low contrast, nuclei extraction was difficult even if the system adopted a color normalization method by using training data of several thousands in different medical facilities. The Supplementary Figure 1 shows the results of nuclear extraction, which is the intermediate process in cytological atypia analysis, and the color distribution of nuclei and the other areas in two institutions. The nuclear extraction was successful in Tokyo hospital, and was not successful in East hospital. Since color distribution of nuclei and other areas had large overlap in East hospital, the nuclear extraction process based on color information was not successful, which resulted in the underestimation of carcinoma.

Lymphoma and carcinoid, which were not epithelial neoplasia, were classified into various classes, probably because such rare subtypes of neoplasia were not trained by using a large amount of data assigned to such classes.

**Figure 4:**
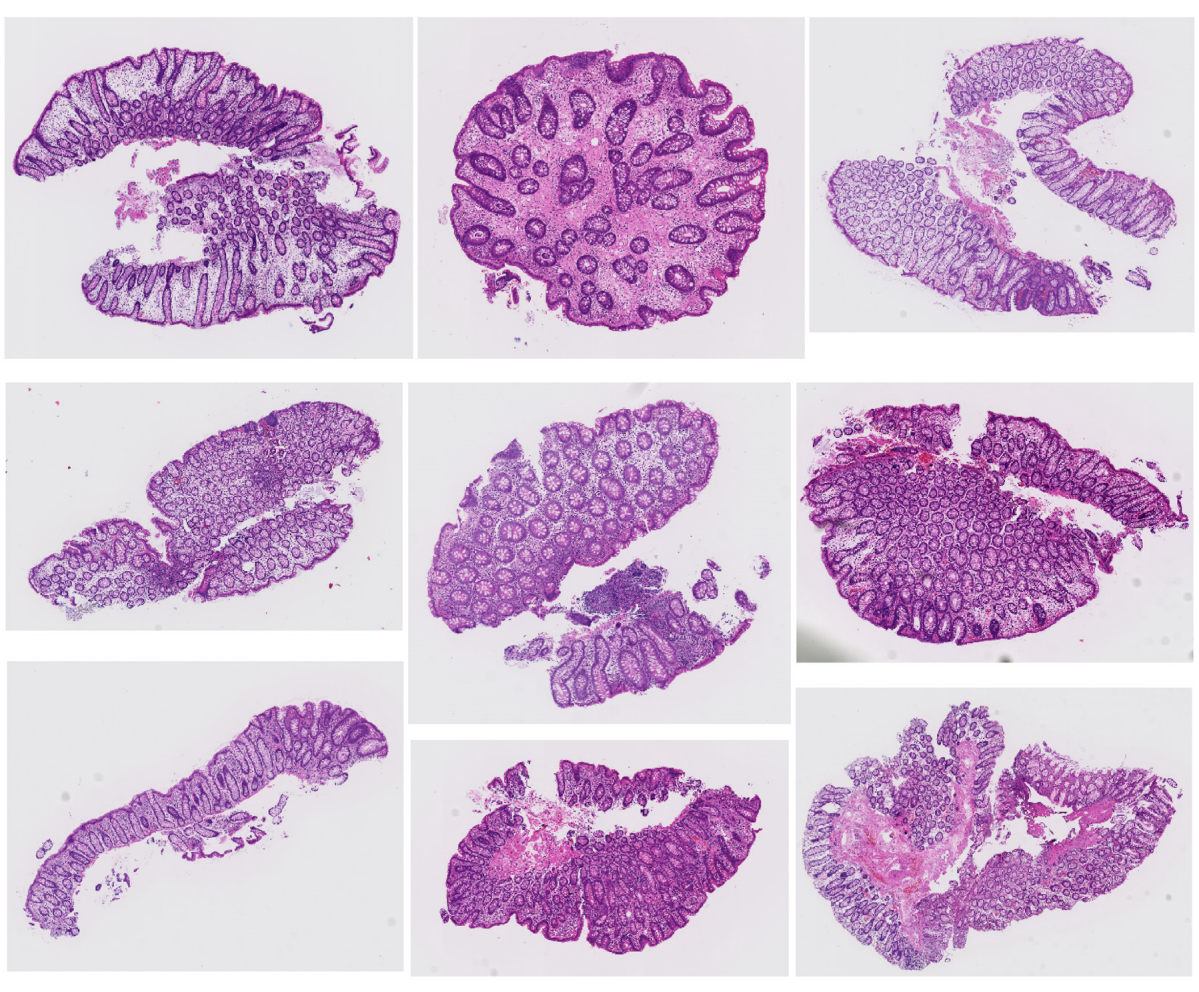
Representative tissues classified as Negative(N) in case a diagnosis by human pathologists is Group 3 on Tokyo hospital

None of the Group 3 tissues were under-classified as Negative(N) on the East hospital dataset, while the 80 out of the 810 Group 3 tissues were under-classified as Negative(N) on the Tokyo hospital dataset. The representative tissue images are shown in Figure 4. Most of them are low grade adenoma, and no tissues with high grade adenoma were underestimated. There are reports concerning inter-observer agreement of human pathologists in the diagnosis of adenoma and non-adenoma. Because this system was trained based on tissues diagnosed by several pathologists in different medical facilities, the system might not classify kinds of tissues with thin glands shown in Figure 4 as Adenoma(A).

In this study, we evaluated whether the system can achieve spec performance without training and tuning using, by data from the two monitor institutions. The undetected rate of carcinoma was 0.0% and 9.3%, while the undetected rate of adenoma was 9.9% and 0.0% for the two institutions, respectively. This system could not achieve spec performance in the undetected rate of carcinoma in East hospital. The primary factor is the difference in the color properties caused by differing H&E staining conditions. We expect that the difference in the color properties of images can be compensated for by training and tuning using the data from the target institution, or by preparing the slides under specific conditions. We demonstrated the following quality control workflow: a diagnosis by the first human pathologist and the prediction by the system were compared with each other, and the tissues over-predicted by the system were rechecked by a second human pathologist. The amount of re-checking by a second pathologist could be reduced to only 36.1% or 27.1% on the Tokyo hospital, and East hospital datasets, respectively. This rate might seem high. There is somewhat of a tradeoff between false negative rate and false positive rate. A system which classifies nearly all positives correctly usually has high false positive rate. In our system, the risk of underestimation of carcinoma is reduced as much as possible, and hence a certain level of over-estimation is unavoidable. Though it is desirable that the over-estimation rate should be low, this system will contribute to the reduction in the burden of work, of double-checking.

There are, however, some limitations on this system. For example, this system is able to classify a blurred image as blurred, but is not to able to reconstruct a clear image from a blurred one. In this system, some instances cannot be classified properly under certain staining conditions. Especially, the classification of lymphoma or carcinoid is insufficient, and some of the low grade adenoma cases are missed. Currently, it is necessary to evaluate this system at target facilities, and adjustment of this system should be performed if necessary. In conclusion, the present study provided promising results for applying an automated histopathological classification of colorectal biopsy specimens into clinical practice, although there are some limitations and requirements. Analysis of histological images is challenging research that requires interdisciplinary collaborations among pathologists and computer scientists. Such collaboration is necessary to tackle the difficult problem of discovering and interpreting novel patterns in histopathological data. To enhance the system performance, it is important not only for further improvement of machine learning, but also standardization of slide preparation. Although the processes toward medical information and communications technology have a lot of social issues due to medical systems, cultures, and industries, such an automated histopathological image analysis system could assist pathologists in the not too distant future.

## MATERIALS AND METHODS

### Patient selection, tissue section preparation and pathological diagnosis

The study was conducted in accordance with the Declaration of Helsinki, and with the approval of the institutional review board of the National Cancer Center in Tokyo, Japan. This study was performed using all colorectal biopsy specimens and polypectomy specimens up to 10mm obtained from routine patients during a 15-week period from January 19, to April 30, 2015, at Tokyo hospital and East hospital in the National Cancer Center, Japan. In total, 1328 tissue specimens consisting of 1068 slides were subject to the survey. The daily histological slide preparation at both hospitals is as follows: Several biopsy and/or polypectomy specimens per patient were fixed in 10% buffered formalin and embedded in the same paraffin block. A single 4-µm-thick section from each paraffin block was mounted on a glass slide and stained with H&E stain by an automated stainer; at least two experienced gastrointestinal pathologists microscopically examined, and made a pathological diagnosis for each tissue specimen according to the Japanese Group Classification [8], in line with routine clinical practice. All cases with diagnostic discordance were reviewed by multiple pathologists, and diagnostic agreement on all cases was established. The final consensus diagnosis was used in this study.

The Japanese Group Classification is applied only to endoscopic biopsy materials, and is applied only to epithelial tissue. Materials obtained by polypectomy, endoscopic resection, or surgery were not included. Japanese Group Classification is as follows: Group X—inappropriate material for which histological diagnosis cannot be made; Group 1—normal tissue or non-neoplastic lesion; Group 2—material for which diagnosis of neoplastic or non-neoplastic lesion is difficult; Group 3— adenoma; Group 4—neoplastic lesion that is suspected to be carcinoma; and Group 5—carcinoma. To resolve the differences between the conventional Western criteria and the Japanese Group Classification, the Vienna Classification was established to combine the basic concepts of the conventional Western criteria. The revised Vienna classification [9] has been widely accepted and consists of five categories, and corresponds to the Japanese Group Classification as follows: Category 1—negative for neoplasia/dysplasia (Group 1); Category 2—indefinite for neoplasia/dysplasia (Group 2); Category 3—low grade adenoma/dysplasia (Group 3); Category 4.1—high grade adenoma/dysplasia (Group 4); and Categories from 4.2 to 5.2—non-invasive carcinoma to submucosal carcinoma or beyond (Group 5). The Japanese Group Classification and Vienna Classification schemes are shown in Table 1.

### Digital image acquisition and automated image analysis system

During the 15-week evaluation period in this validation study, the following procedures were performed every weekday. Glass slides were anonymized at each of the hospitals, and then converted to a WSI by the NanoZoomer 1.0-HT digital slide scanner (Hamamatsu Photonics K.K., Hamamatsu, Japan) at Tokyo hospital, and the NanoZoomer 2.0-HT digital slide scanner (Hamamatsu Photonics K.K) at East hospital, at 40x (0.23 µm/pixel) magnification. The WSI files of East hospital were encrypted on the server located on site, transferred to the server at Tokyo hospital via network, and decrypted, all completely automatically. The system automatically detects WSI files of both Tokyo hospital and East hospital, and starts analyzing the detected WSIs. All aspects concerning the pathological diagnoses by the pathologists were undisclosed to researchers and statisticians during this period.

### Automated colorectal classification scheme and image analysis

**Figure 5:**
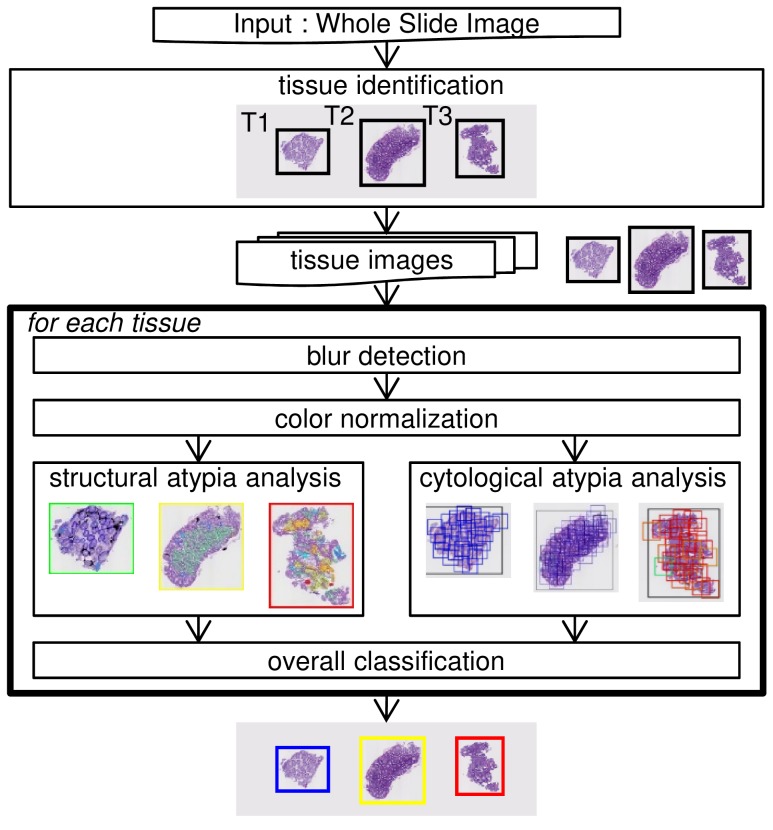
Structure of the automated colorectal classification scheme

The whole structure of our protocol is shown in Figure 5. When a WSI is first accepted, each tissue region is identified one by one from the low-resolution image of whole area. Following this, the corresponding high-resolution images get retrieved from the WSI. Then, the following processes are performed for each tissue image: blur detection, color normalization, cytological atypia analysis, structural atypia analysis, and overall classification. Finally, the image gets classified into one of the four categories, either: suspicious for carcinoma (Positive), suspicious for adenoma (Adenoma), no neoplasia (Negative), or unclassifiable (Unclassifiable) for each tissue. The similarities between the revised Vienna classification, the Japanese group classification, and the final output of our system are summarized in Table 1. The classifier in each of the six processes had been trained using the training set, and its parameters had been tuned using the validation set, in advance. The system had not been modified using the data derived from either Tokyo hospital or East hospital. The details of our approach are described in the following subsections, which correspond with each of the steps.

### Blur detection

Blurred regions, which occur when the lens is out-of-focus, can practically lead to serious problems downstream, because these blurred regions have unpredictable effects on image segmentation and other quantitative image features. Therefore, we used a blur detection method in order to rule out the possibility of an erroneous classification. Our procedure are present in the Supplementary Material S1.

### Color normalization

The variation in protocols for preparing histological sections and digitizing the images inevitably results in batch effects, especially image color, which is a critical aspect that influences image processing. Therefore, we used a color normalization method that converted each color image into one that was free from batch effects. Our procedure are present in the Supplementary Material S2.

### Structural atypia analysis

The degree of cytological atypia and structural atypia contributes greatly to the diagnostic process for colorectal neoplasia. The distinction between adenoma and well- or moderately differentiated adenocarcinoma depends largely on the evaluation of structural atypia. Therefore, we proposed a method that quantifies the degree of gland changes corresponding to structural atypia, and classifies a tissue image into one of four classes: low atypia level, middle atypia level, high atypia level, or unclassifiable. Our method for structural atypia analysis was designed by the desire to mimic a pathologist, and, we provided methods putting an emphasis on easily understandable and acceptable bases for pathologists instead of black-box learning approaches. Our procedure was roughly as follows: (Step 1) a mask image was created as pre-processing. (Step 2) Nuclear regions of cells that make up glands were extracted using image processing methods from the tissue image on a low magnification, and the thickness of each extracted glandular nuclear component was quantified. (Step 3) When glandular components with thick cells were found by the above Step 2, nuclear and cytoplasmic regions of the cells making up the glands were extracted using image processing methods from the tissue image, on a high magnification, and the arrangement of the nuclei and cytoplasm in each extracted gland component was evaluated. We designed the two-step approach, which uses low-magnification analysis and, and then high-magnification analysis as necessary, to reduce the computational time. The details of our steps are described in the Supplementary Material S3.

**Figure 6:**
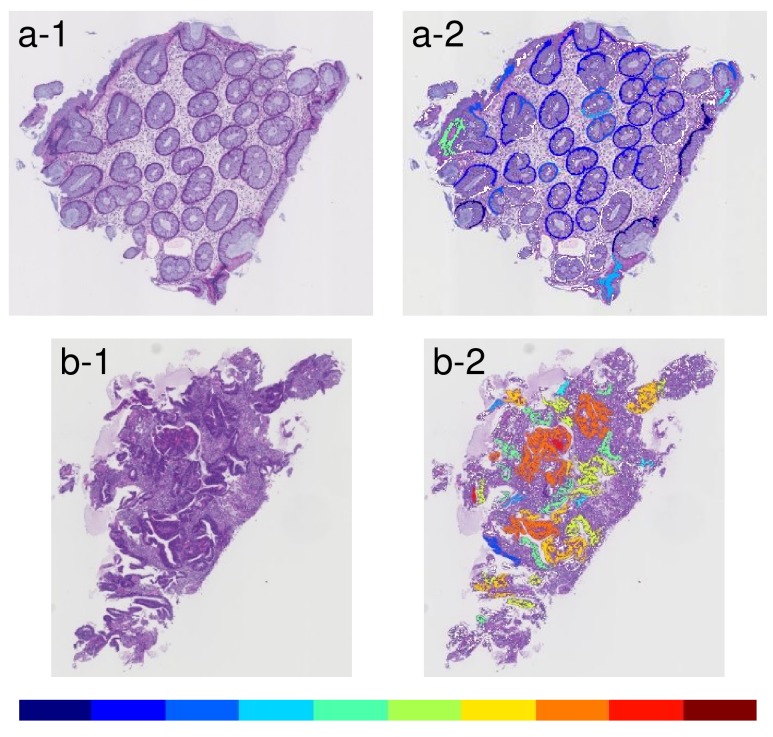
Examples of results that indicated glandular level by ten different colors between cool and warm (a-1) An example of an input image with low grade atypia. (a-2) Visualization-displayed glandular level of the input image a-1. (b-1) An example of an input image with high grade atypia. (b-2) Visualization-displayed glandular level of the input image b-1. Warm colors indicate a thicker object and cool colors indicate a thinner object.

The glandular level was visualized by 10 colors, which changed gradually from cool to warm colors. The coolest color indicates gland level near 1 (i.e. thinner), while the warmest one indicates gland level near 10 (i.e. thicker). The results of Step 2 are shown in Figure 6.

**Figure 7:**
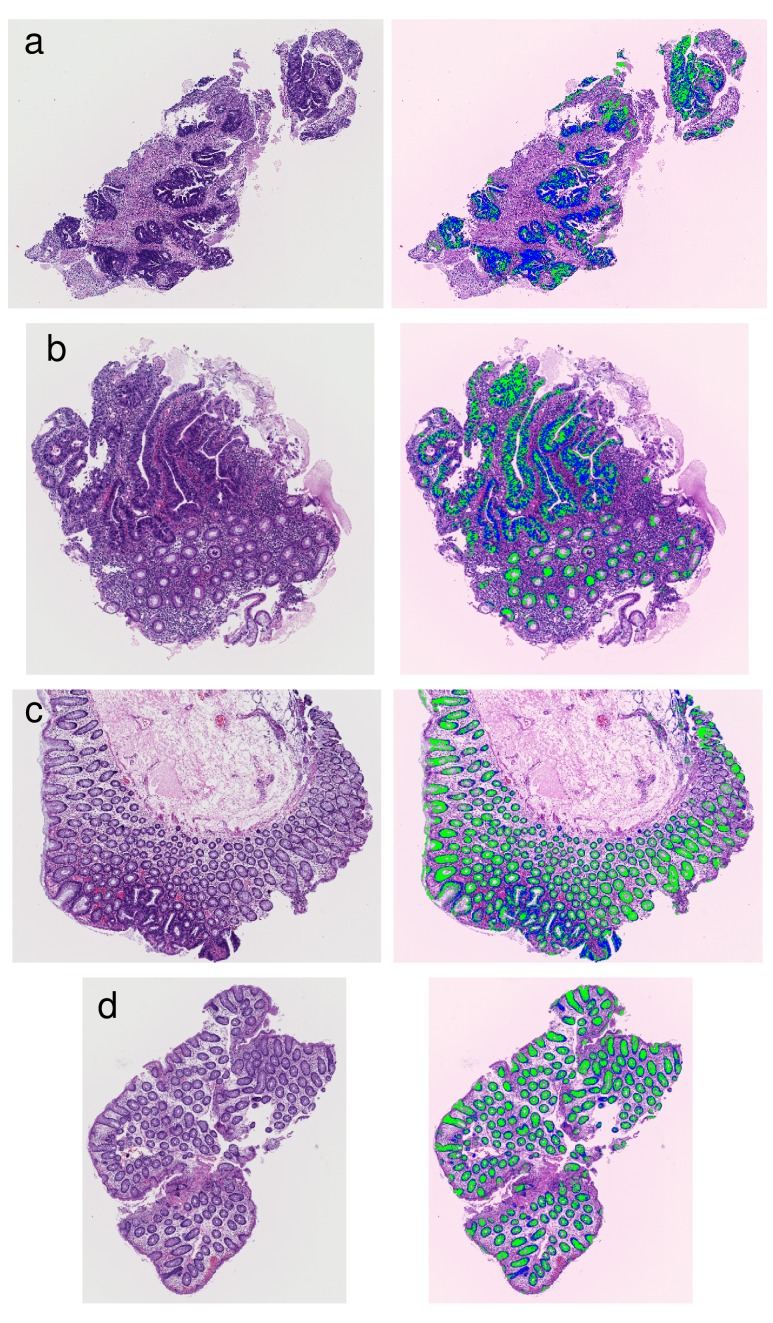
Examples of results that segmented glandular nuclei and glandular cytoplasm (a) An example of high grade atypia. (b) An example including high grade atypia. (c) An example including intermediate grade atypia. (d) An example of low grade atypia. The extracted glandular nuclei are shown in blue, and the extracted glandular cytoplasms are shown in green.

As illustration of Step 3, four examples of the segmentation of glandular nuclei and glandular cytoplasm are shown in Figure 7.

### Cytological atypia analysis

The diagnosis of poorly differentiated adenocarcinoma depends on the degree of cytological atypia. Analysis of cytological atypia used a method [10], which co-authors at NEC Corporation previously developed for classifying gastric cancer. This method is based on the multi-instance learning (MIL) [11] using a neural network [12]. Our procedure was as follows: First, regions of interest (ROIs) were formed so as to cover an entire tissue image using the density of nuclei. Second, the degree of tissue change was computed in a range from 0 to 1, using a MIL classifier mentioned above for each ROI. Finally, the input tissue image was classified into one of two classes: high atypia level or low atypia level according to a threshold of the mean-square of top three values computed for each ROI. The threshold was adjusted to avoid missing poorly differentiated adenocarcinoma on a training data set from a different medical facility. Supplementary Figure 2 shows extracted nuclei superimposed on ROI, and a result tissue image with colors according to value from 0 to 1 computed for each ROI.

### Overall Classification

Overall Classification categorized a tissue section into one of four classes: Positive, Adenoma, Negative, or Unclassifiable, according to the decision result (high, middle, or low) of structural atypia analysis and the result of cytological atypia analysis (high or low). The result with the higher atypia level was adopted preferentially.

### Evaluation method

Our system was developed as a quality control system to avoid overlooking of carcinoma in the diagnosis process. Currently, two pathologists independently make a diagnosis for the entire tissue sections. Instead of 100% manual double-checking, the quality control system could be considered in the following: the diagnosis by a pathologist and the prediction by the system are compared with each other, and the tissues that were over-predicted by the system are rechecked by a pathologist. It is possible to reduce the incidence of false diagnosis without double-checking entire tissue sections. In such a quality control system, the false negative rate (cancer tissue being detected as non-cancer) should be suppressed as much as possible, while the false positive (non-cancer being detected as cancer) is tolerable. To evaluate the performance of the system, we adopted the following three indices: undetected rate of carcinoma, undetected rate of adenoma, and over-detected proportion to total, instead of the accuracy, which is the percentage of correctly predicted labels over all predictions. The three indices are as follows:

$$\text{undetected rate of carcinoma}=\frac{n\left(A|G5\right)+n\left(N|G5\right)}{n\left(G5\right)}\times 100$$

$$\mathrm{undetected rate of adenoma}=\frac{n\left(N|G3\right)}{n\left(G3\right)}\times 100$$

$$\mathrm{over-detected proportion}=\frac{n\left(P|G3\right)+n\left(P|G1\right)+n\left(A|G1\right)+n\left(X|G3\right)+n\left(X|G1\right)}{n\left(ALL\right)}\times 100$$

where n(*a*|*b*) is the number of tissue examples, in case that a diagnosis by human pathologists is *b* and a prediction by the system is *a*. An index called the “over-detected proportion” is equivalent to the proportion of second checking, and is directly related to various costs. The undetected rate of carcinoma and undetected rate of adenoma are expected to be almost zero, while keeping the over-detected proportion low. Note that there is a trade-off relation between “undetection” and over-detection.

Our system was taught through training data of several thousands in different medical facilities, and was tuned to satisfy the above requirements using validation data. The details of the validation data were 15% of G5, 35% of G3, and 50% of G1. Our system was tuned so as to have the following performance: the undetected ratio of carcinoma ≈1%, the undetected ratio of adenoma ≈5%, and the over-detected proportion ≈30%.

## SUPPLEMENTARY MATERIALS FIGURES


