# Supplementary material for: Automated histological classification of whole slide images of colorectal biopsy specimens
Source: Oncotarget. 2017 Oct 12;8(53):90719–29. doi: 10.18632/oncotarget.21819 (PMC5710880; doi:10.18632/oncotarget.21819)
Supplement: Supplementary file 1 [file oncotarget-08-90719-s001.pdf]

Automated histological classification of whole slide images of colorectal biopsy specimens

SUPPLEMENTARY MATERIALS

Supplementary Material 1. Blur detection

Our procedure was as follows: first, a grayscale image corresponding to the degree of blurriness was generated by using the method based on the Haar wavelet transform [1]. Second, the grayscale image was converted into a binary image, where blurred regions were white, according to a predetermined threshold. Finally, whether a given tissue image was blurred or not, was determined by the area ratio of the white-pixel regions of the converted binary image, to the total tissue region. Supplementary Figure S3 shows two examples of detected blurred images.

Supplementary Material 2. Color normalization

Our procedure was as follows: first, regions of nuclei and background were roughly extracted from an image according to color and shape, and the average RGB color of the nuclear regions, and the background were computed. Second, in order to transform the computed average color of the nuclear regions and background to the specific predefined nuclear and white color respectively, a transformation curve was computed for each of the RGB components. This curve was an adopted gamma curve. Finally, the input RGB color image was corrected using the transformation curve calculated above. This method can be applied to various image datasets collected during experimental setups. Supplementary Figure S4 shows an example of color normalization and color distribution.

Supplementary Material 3. Structural atypia analysis

In pre-processing (Step 1), the mask image was created to eliminate non-target areas such as lymph nodule and crushed artifact. We proposed a method for automated segmentation of lymph nodules. The algorithm involved three steps: enhancement of hematoxylin color, binarization, and dilatation of the binary image to expand the extracted objects corresponding to lymph nodules.

In low-magnification analysis (Step 2), the thickness of glandular nuclei was quantified to evaluate glandular atypia based on the low-magnification image, 1.25x, where a thickness of normal glandular nuclei is approximately one pixel. We proposed a method for quantifying thickness of glandular nuclei, which would correspond to glandular atypia level. The algorithm involved eight steps: (1) reduction of image size using bicubic interpolation [2], (2) conversion of RGB values (24-bit) into grayscale values (8-bit), (3) suppression of the low maxima to remove less significant objects [3], (4) enhancement of contrast using morphological operations [4], (5) binarization, (6) deletion of the regions superposed on the lymph nodules extracted in Step 1, (7) calculation of the glandular level value lv in the range of 1 to 10 for each glandular nuclei component. The level value was calculated as $lv = \min\{\max\{\lfloor 20 * R_S + 0.4 * R_L \rfloor - 1, 1\}, 10\}$, where $R_S = S_o / S_{bb}$, $R_L = S_o / L_{bb}$, S_o is area of a object, S_{bb} is area of bounding box, L_{bb} is perimeter of bounding box, and (8) classification of thin-class or thick-class for an input tissue image based on a rule-base classifier using the following features: the number of objects per level, cumulative area of objects per level, and maximum area of objects per level. In the case where a tissue image is classified as thick-class, the process proceeded to the next high-magnification analysis (Step 3). Two examples of the image processing for glandular segmentation are shown in Supplementary Figure S5.

In high-magnification analysis (Step 3), the local arrangement of glandular components was evaluated. We proposed a method for segmenting glandular nuclei, cytoplasm, lumen, and stroma, and evaluating the distribution of both glandular nuclei, and cytoplasm. The algorithm involved six steps: (1) segmentation of a tissue image into glandular nuclei, and glandular cytoplasm including goblet cells, lumen, and stroma by using color information, (2) extraction of glandular components combining glandular nuclei, and cytoplasm, (3) deletion of the regions superposed on the lymph nodules extracted in Step 1, (4) calculation of six features for each gland, f1: the number of nuclear components, f2: the ratio of nuclear component area to a glandular area, f3: the average area of the nuclear components (nuclear components area/the number of nuclei components),

f4: the ratio of the number of nuclear components to a glandular area, f5: the ratio of the area of nuclear components including lumen side half of gland thickness, to that of the included stroma side half of the glandular thickness using lumen location (Supplementary Figure S6), f6: the ratio of the area of nuclear components including lumen side half of glandular thickness, to that of the included stroma side half of the glandular thickness using stroma location (Supplementary Figure S6), (5) classification of disturbed-class, middle-class, or regular-class for each gland based on the rule which was constructed by the rule-base classifier, and was modified under the consideration of the morphological meaning, and (6) classification of high atypia level, middle atypia level, or low atypia level for an input tissue image, when even one disturbed-class, middle-class, or otherwise is present, respectively.

REFERENCES

1. Tong H, Li M, Zhang H, Zhang C. (2004). Blur detection for digital images using wavelet transform. Multimedia and Expo, 2004 ICME'04 2004 IEEE International Conference on: IEEE), pp. 17-20.
2. Keys R. Cubic convolution interpolation for digital image processing. IEEE transactions on acoustics, speech, and signal processing. 1981; 29: 1153-60. doi:
3. Soille P. (2013). Morphological image analysis: principles and applications: Springer Science & Business Media).
4. Serra J. (1982). Image analysis and mathematical morphology, v. 1: Academic press).

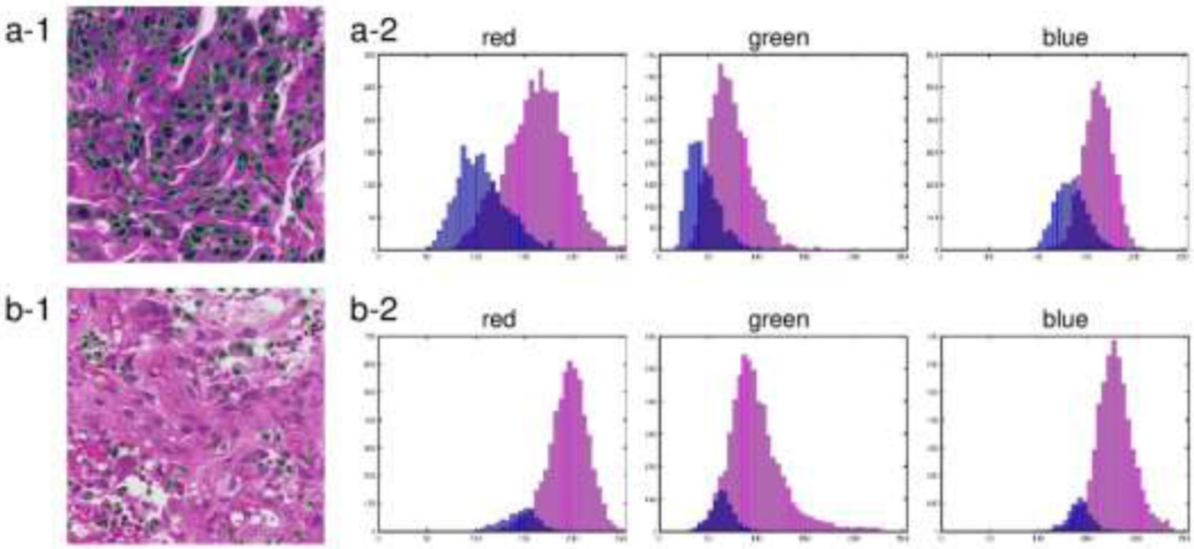

Supplementary Figure 1: Example results of nuclear extraction and histograms of nuclei and the other areas on images from Tokyo hospital and East hospital. (a-1) Successful example of nuclei extraction in Tokyo hospital (shown in green). (b-1) Histogram of nuclei (shown in blue) and the other areas (shown in pink) on image from Tokyo hospital. (a-2) Fail example nuclei extraction in East hospital (shown in green). (b-2) Histogram of nuclei (shown in blue) and the other areas (shown in pink) on image from East hospital.

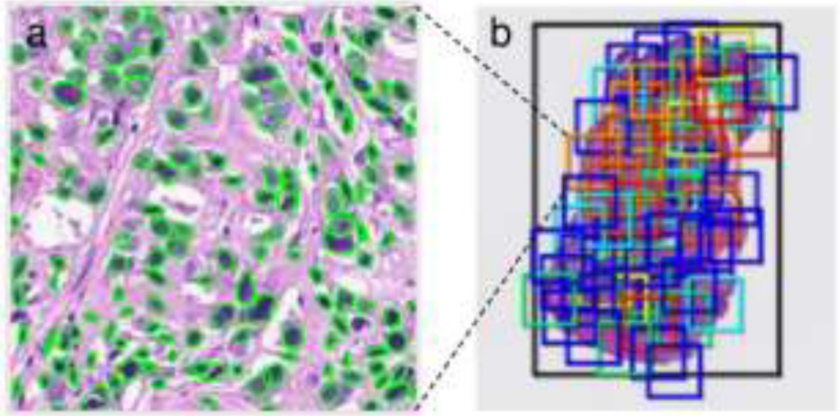

Supplementary Figure 2: An example of results of cytological analysis. (a) An enlarged ROI of image-b (right figure) where extracted nuclei shown in green were superimposed on the ROI. (b) A result tissue image colored according to value from 0 to 1 computed for each ROI.

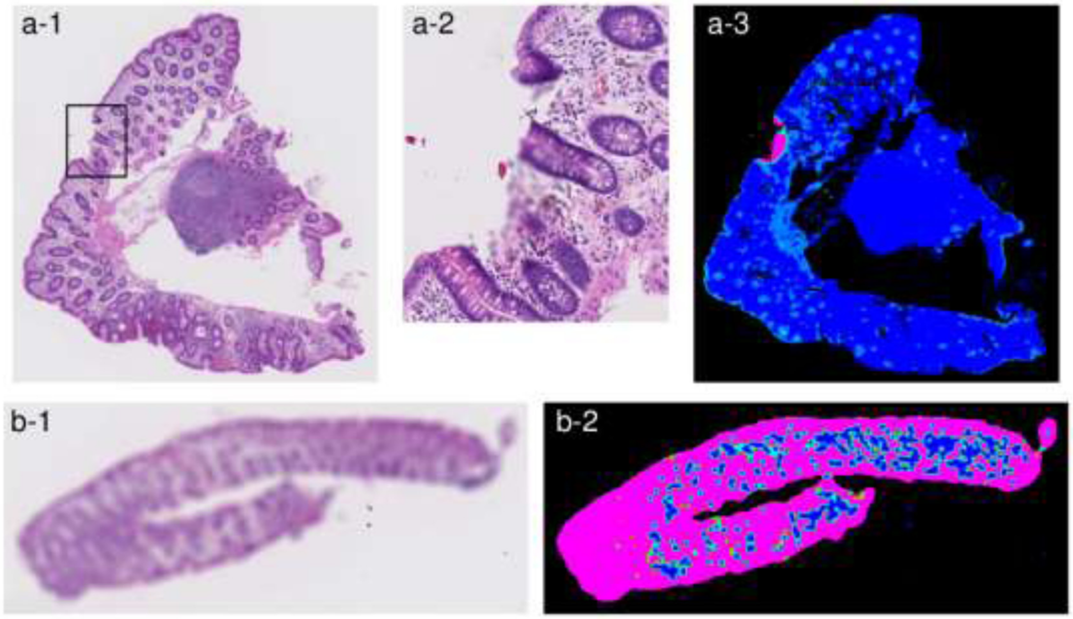

Supplementary Figure 3: Examples of detected blurred image. (a-1) Input tissue image. (a-2) Image with an enlarged portion of image-a. (a-3) Detected blur regions shown in pink color. (b-1) Input tissue image. (b-2) Detected blur regions shown in pink color.

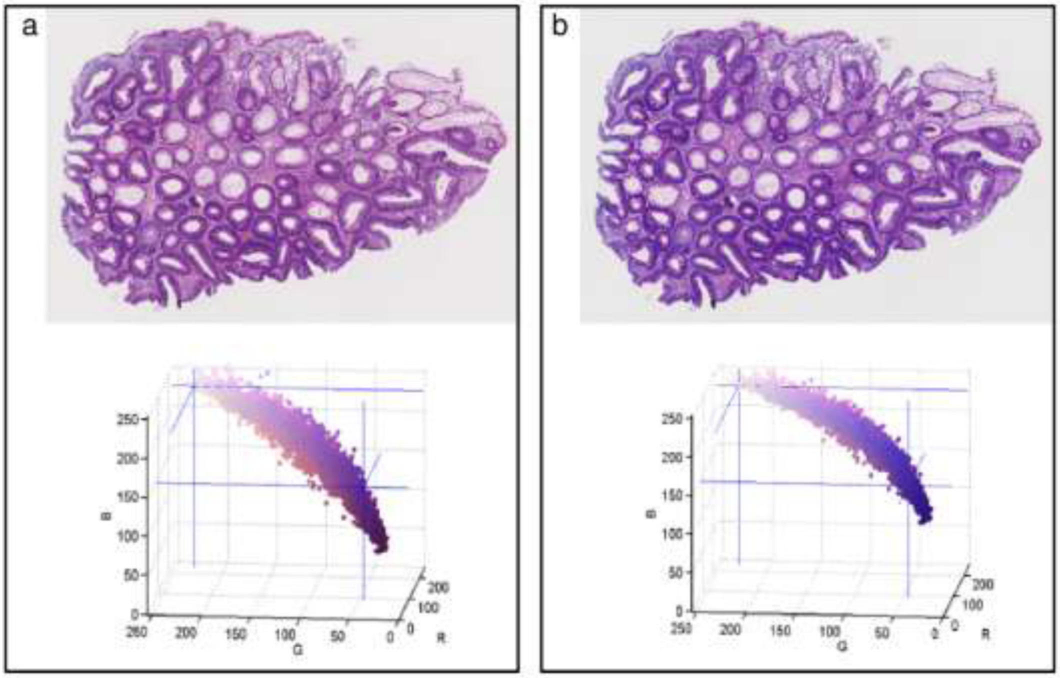

Supplementary Figure 4: An example of color normalization. (a) Image and color distribution before color normalization. (b) Image and color distribution after color normalization.

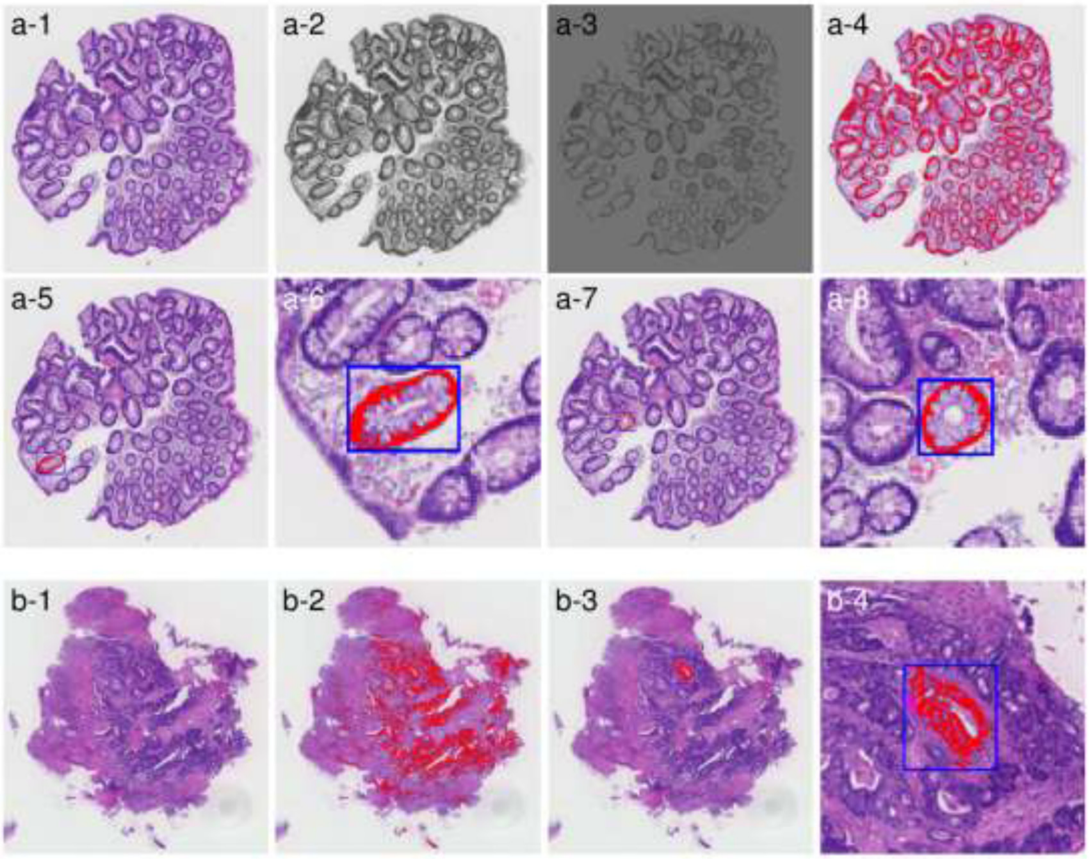

Supplementary Figure 5: Process of the proposed method for glandular nuclei segmentation at low-magnification image. (a-1) Original image at 1.25x. (a-2) Image converted to grayscale. (a-3) Image with a suppressed low maxima. (a-4) Extracted glands after enhancement and binarization. The extracted glands shown in red were superimposed on the original image. (a-5) An object and its bounding box. (a-6) Image with an enlarged portion of image a-5. The blue square shows the bounding box of the object. The computational values are $R_L = 1.67$ and $R_S = 0.26$. (a-7) An object and its bounding box. (a-8) Image with an enlarged portion of image a-7. The computational values are $R_L = 1.23$ and $R_S = 0.23$. (b-1) Original image at 1.25x. (b-2) Extracted glands. The extracted glands shown in red were superimposed on the original image. (b-3) An object and its bounding box. (b-4) Image with an enlarged portion of image b-3. The blue square shows the bounding box of the object. The computational values are $R_L = 2.87$ and $R_S = 0.35$.

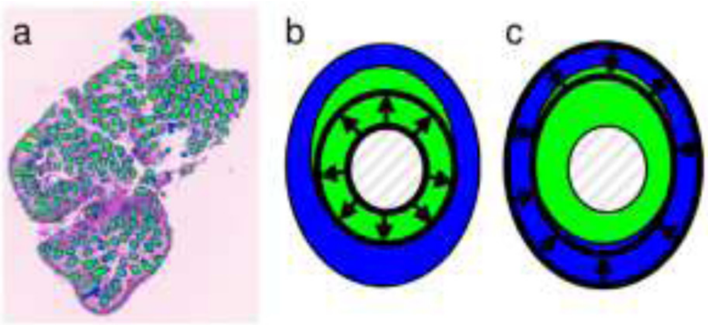

Supplementary Figure 6: Illustrations of features f5 and f6. (a) Example of result that segmented glandular nuclei (shown in blue) and glandular cytoplasm (shown in green). (b) Illustration of f5. A half position of the glandular thickness can be specified by dilating lumen region. (c) Illustration of f6. A half position of the glandular thickness can be specified by eroding glandular region from stroma side.
